# Sutureless jejuno-jejunal anastomosis in gastric cancer patients: a comparison with handsewn procedure in a single institute

**DOI:** 10.1186/1471-2482-12-S1-S27

**Published:** 2012-11-15

**Authors:** Luigi Marano, Bartolomeo Braccio, Michele Schettino, Giuseppe Izzo, Angelo Cosenza, Michele Grassia, Raffaele Porfidia, Gianmarco Reda, Marianna Petrillo, Giuseppe Esposito, Natale Di Martino

**Affiliations:** 1 Institution: VIII General and Gastrointestinal Surgery (Chief Prof. N. Di Martino) - School of Medicine - Second University of Naples - Piazza Miraglia 2, 80138 Naples, Italy

## Abstract

**Background:**

The biofragmentable anastomotic ring has been used to this day for various types of anastomosis in the gastrointestinal tract, but it has not yet achieved widespread acceptance among surgeons. The purpose of this retrospective study is to compare surgical outcomes of sutureless with suture method of Roux-and-Y jejunojejunostomy in patients with gastric cancer.

**Methods:**

Two groups of patients were obtained based on anastomosis technique (sutureless group versus hand sewn group): perioperative outcomes were recorded for every patient.

**Results:**

The mean time spent to complete a sutureless anastomosis was 11±4 min, whereas the time spent to perform hand sewn anastomosis was 23±7 min. Estimated intraoperative blood loss was 178±32ml in the sutureless group and 182±23ml in the suture-method group with no significant differences. No complications were registered related to enteroanastomosis. Intraoperative mortality was none for both groups.

**Conclusions:**

The Biofragmentable Anastomotic Ring offers a safe and time-saving method for the jejuno-jejunal anastomosis in gastric cancer surgery, and for this purpose the ring has been approved as a standard method in our clinic. Nevertheless currently there are few studies on upper gastrointestinal sutureless anastomoses and this could be the reason for the low uptake of this device.

## Background

The concept of compression anastomosis was introduced for the first time in February 1826 at the meeting of the Societe Royale de Medicine de Marseilles by Felix-Nicholas Denans who performed an end-to-end anastomosis using a metallic (silver or zinc) ring in a canine model [1]. At that time, this technique was still evolving, and in 1892 Murphy developed a new device of compression anastomosis in humans [2-6], which has been called “Murphy’s button”, that was extensively used. However, its clinical success was limited for relatively common anastomotic stenosis [7]. Approximately one century after Murphy, in 1985, Hardy et al [8] described the biofragmentable anastomotic ring (BAR). This device has been used so far for various types of anastomosis in the upper and lower gastrointestinal tract [6-14], for elective and emergency surgery [8,10-18], but it has not yet achieved widespread acceptance among surgeons [19]. The purpose of this retrospective study is to compare surgical outcomes of BAR with suture method of Roux-and-Y jejunojejunostomy in patients with gastric cancer who have undergone to total or partial gastrectomy.

## Material and methods

**Figure 1:**
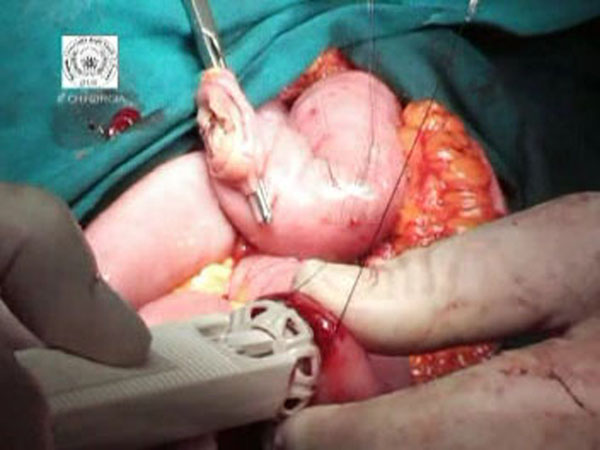
Introduction of 28mm BAR into the proximal jejunum

**Figure 2:**
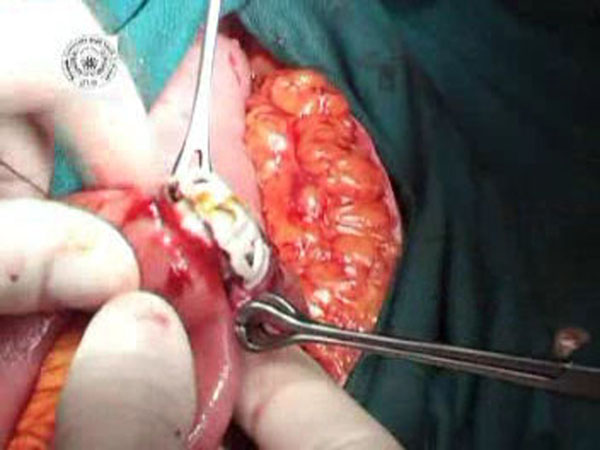
The other side of the BAR is inserted into the end jejunal wall

**Figure 3:**
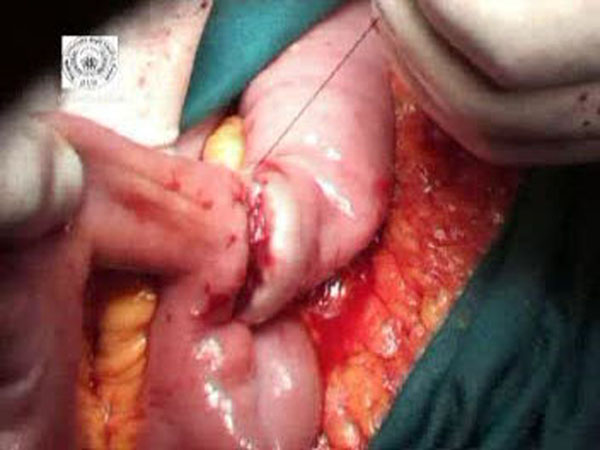
The second purse-string is tied

**Figure 4:**
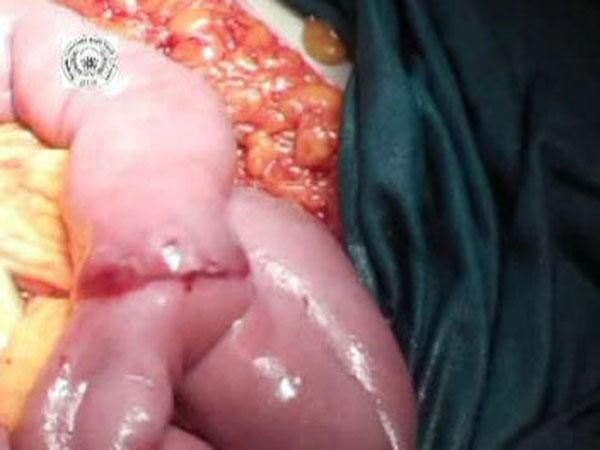
The BAR is snapped forming a serosa-to-serosa inverted sutureless anastomosis

From April 2002 to June 2010, 131 patients with a mean age of 64 years (range 37-89), 87 males and 44 females with a diagnosis of gastric cancer referred to the 8th General and Gastrointestinal Surgery of the Second University of Naples. Six of these 131 patients (3 males and 3 females) were not resectable in the course of surgery due to local extent of the tumor; one patient was not operable due to the presence of restrictive lung disease and aortic aneurysm, and one refused the operation. The patients who underwent gastric surgery were 123 (82 males and 41 females). 112 patients had a diagnosis of gastric adenocarcinoma, 10 non-Hodgkin lymphoma and 1 gastric carcinoid. Two groups of patients were obtained based on anastomosis technique: in the first group of 64 patients (43 males and 21 females, mean age 64.9) an end-to-side Roux-and-Y jejunojejunostomy was performed using a BAR after 57 total gastrectomy and 7 gastric resections. In the second group of 59 patients (37 males and 22 females, mean age 63.95) an end-to-side Roux-and-Y jejunojejunostomy suture method anastomosis was performed after 57 total gastrectomy and 2 gastric resections. BAR is made of 2 identical rings, each composed of 87.5% absorbable polyglycolic acid and 12.5% barium sulfate acting as a “radiopaque dye” to enhance x-ray imaging (abdominal X-ray examination showed BAR fragmentation approximately between 2 and 3 weeks after surgery [20]) . The rings have an internal lumen that varies from 11 to 20 mm in diameter, depending on the size and are placed into the cut bowel ends. When the device is closed a 1.5- to 2.5-mm gap remains between the 2 rings to prevent extensive tissue ischemia. The appropriate size of BAR device is crucial for a successful anastomosis; the ring must be compatible with the diameter of the bowel and the thickness of the bowel wall [17-19]. If the gap between the 2 rings is too large, a proper seroserosal approximation of the bowel ends will not be achieved, whereas if the compression zone is too narrow, the closing dynamics of the BAR can be altered and the tissue grasped in the gap can be subjected to extensive ischemic necrosis, leading to early detachment of the BAR [21]. An external diameter of 28 mm was preferred in all our patients for enteric anastomosis, whereas that of 31 mm or more was used in colonic or rectal anastomosis [19]. During the procedure, excessive snap pressure should be avoided since the BAR material is relatively friable [18]. The BAR anastomosis was performed by using a standard technique: after a total gastrectomy with an end-to-side esophagojejunostomy or a partial gastrectomy with side-to-end gastrojejunostomy, monofilament not- absorbable pursestring suture is placed, before bowel resection, at the jejunal wall along the pursestring clamp applied tangentially to antimesenterical fold, approximately 60 cm down from esophagojejunostomy or gastrojejunostomy. After bowel resection, a BAR of 28 mm is introduced into the proximal jejunum first by means of the inserter and then the pursestring suture is tied (Figure 1). After removal of the inserter, the other side of the BAR is inserted into the end jejunal wall and the second pursestring is tied (Figures 2 and 3). The BAR is snapped shut by index finger and thumb pressure of two hands, forming a serosa-to-serosa inverted sutureless anastomosis (Figure 4). Before closing the ring the possible rotational error at the anastomosis is corrected. The manually sutured jejunojejunal anastomosis, following the same procedures as described for suturless anastomosis, is achieved by continuous 3-0 polyglycolic acid multifilament in two layers with inversion technique. Patient demographics, operative procedure, type and location of the anastomosis, overall operating time, intraoperative blood loss, postoperative course and complications, if observed, were recorded for every patient. Postoperative completeness of the BAR anastomosis and fragmentation of the BAR ring were confirmed by abdominal x-ray at 7^th^ and 30^th^ day after surgery. The study was approved by the ethics committee of Second University of Naples and conducted according to the ethical standards of the Helsinki declaration. Each patient gave informed written consent.

## Results

64 end-to-side Roux-and-Y jejunojejunostomy were performed using a BAR after 57 total gastrectomy and 7 gastric resections. 59 end-to-side Roux-and-Y jejunojejunostomy suture method anastomosis were performed after 57 total gastrectomy and 2 gastric resections for gastric cancer. The mean time spent to complete a BAR anastomosis was 11±4 min, being the time spent to perform hand sewn anastomosis 23±7 min (p=0.030 ). In the BAR group the estimated operative blood loss was lower compared to the suture-method group (178±32ml and 182±23ml respectively), however the difference didn’t reach a statistical significance (p=0.065). The postoperative course was uneventful in 75% (n=44) patients in the suture group and in 95.1% (n=61) the patients in the BAR group. No intraoperative mortality for both groups was found. The assessment of surgical morbidity revealed a complication rate of about 7.8% for the compression anastomosis group (2 intestinal obstructions treated with surgery; 1 duodenal fistula treated with medical therapy; 2 wall infections treated with medical therapy) compared to 8.5% for the sutured anastomosis group (1 duodenal fistula treated with medical therapy; 1 pancreatic fistula treated with medical therapy; 1 intestinal obstruction treated with surgery; 2 esophago-jejunal anastomotic leakages, respectively, treated with medical and surgical therapy) even if the complications are independents of the enteroanastomosis. The non-surgical morbidity was 16.8% for the BAR group and about 14.1% for the hand sewn group (p=0.183). No significant differences were noted between the groups in the starting time to oral feeding and intestinal canalization (Table 1). The duration of the postoperative hospital stay was also similar in both groups (10±2days in the BAR group; 10±3 in the suture group; p=0.137).

**Table 1:** Perioperative outcomes of 64 compression end-to-side Roux-and-Y jejunojejunostomy and 59 handsewn end-to-side Roux-and-Y jejunojejunostomy

	Compression anastomosis (n=64)	Handsewn anastomosis (n=59)	p
Estimated intraoperative blood loss	178±32 ml	182±23 ml	.065
Mean jejunojejunostomy time	11±4 min	23±7 min	<0.05
Intraoperative mortality	0%	0%	N.E.
Surgical morbidity	7.8%	8.5%	N.E.
Intestinal obstruction	2 (n)	1 (n)	N.E.
Duodenal fistula	1 (n)	1 (n)	N.E.
Pancreatic fistula	/	1 (n)	N.E.
Esophago-jejunal leakage	/	1 (n)	N.E.
Wall infections	2 (n)	/	N.E.
Non-surgical morbidity	16.8%	14.1%	.183
Starting time to oral feeding	7^th^ day	7^th^ day	/
Intestinal canalization	2.1±0.6 min	2.3±0.5 min	.321
Mean hospital stay	10±2 days	10±3 days	.137

N.E. Not evaluated

## Discussions

The usefulness of BAR is well established in colonic anastomoses, but the effectiveness of a compression ring in small bowel anastomoses after gastric cancer surgery has not yet been well proven. Encouraged by little but favorable experiences with the device in colonic surgery we decided to analyze the outcomes of jejuno-jejunal BAR anastomosis compared with jejuno-jejunal hand sewn anastomosis. Our results demonstrate that patients with a jejuno-jejunal BAR anastomosis recover from upper gastrointestinal resections with no delay when compared to those with a manually sutured, conventional anastomosis. The most significant complication associated with anastomosis is anastomotic leakage [19]: although the occurrence of severe complications was lightly more frequent in the suture group (8.5%) when compared with sutureless group (7.8%), they were independent of the enteroanastomosis. In particular, the none overall jejuno-jejunal leak rate in the present study, as exhibited also by other Authors (2-4.2%) [10-14,17,18,22,23], probably indicate that the compression anastomosis is effective and a safe surgical procedure. Furthermore the surgical technique of BAR anastomosis represents a standardized approach with a very low period of the learning curve. Selection of the appropriate size of the ring and gap width is thought to be one of the critical determinants for a successful BAR anastomosis [19]. In the present study, for ease of use, we preferred to use the ring with external diameter of 28 mm. without any resistance at introduction into bowel lumen for all patients. Another advantage of BAR anastomosis is that it is a faster procedure than hand sewn method, because the mean time of compression procedure is approximately 50% less than the suture procedure, as resulting from our data (11±4 min of BAR anastomoses versus 23±7 min of suture anastomoses (p<0.05)). Therefore it can be applied more preferably to patients with comorbidities where both rapidity and security of the anastomosis is required [14,16,17,22,23].

## Conclusions

In our opinion the Biofragmentable Anastomotic Ring offers a safe and time-saving method for the jejuno-jejunal anastomosis in gastric cancer surgery, and for this purpose the ring has been approved as a standard method in our clinic. Nevertheless, currently there are few studies on upper gastrointestinal BAR anastomoses and this could be the reason for the low uptake of this device.

## List of abbreviations used

BAR: Biofragmentable Anastomotic Ring.

## Competing interests

Authors have no conflicts of interest or financial ties to disclose.

## Authors' contributions

All authors approved the final manuscript. *Study concept and design*: NDM and LM; *Acquisition of data*: BB, MS, RP; *Analysis and interpretation of data*: AC, LM, GI, GR, MP; *Drafting of the manuscript*: LM, BB; *Critical revision of the manuscript for important intellectual content*: NDM, LM, GI; *Statistical analysis*: MG, GE, AC; *Study supervision*: NDM, LM.
